# An Immunodeficiency Disorder Presenting in the Neonatal Period

**DOI:** 10.7759/cureus.59374

**Published:** 2024-04-30

**Authors:** Hadi M Fakih, Abdallah Dbouk

**Affiliations:** 1 Pediatrics, Faculty of Medical Sciences, Lebanese University, Beirut, LBN; 2 Pediatrics, Sheikh Ragheb Harb University Hospital, Toul, LBN; 3 Pediatrics, Faculty of Medicine, Lebanese University, Beirut, LBN

**Keywords:** fungi, bacteria, phagocytosis, dhr, nbt, cgd, immunodeficiency, abscess, newborn

## Abstract

Primary immunodeficiency (PID) Disorders include a variable group of diseases that are classified according to the functional defects encountered. Chronic granulomatous disease (CGD) is inherited as an X-linked recessive disorder in many cases, and it is the clinical model of disorders of phagocytosis. Skin and solid organs abscesses are the most common presenting symptoms; we will report the case of a four-day-old boy admitted to our hospital for a neck mass with purulent discharges associated with umbilical stump and circumcision site infection; the diagnosis of CGD was later confirmed by the Dihydrorhodamine (DHR) test that turned out to be positive.

## Introduction

Inborn errors of immunity (IEIs) have a wide range of manifestations, chronic granulomatous disease (CGD) represents the classic disorder of phagocytosis due to a deficiency in one of the subunits (GP91Phox, P47Phox, P40Phox, P67Phox, P22Phox) of the nicotinamide adenine dinucleotide phosphate (NADPH) oxidase complex deficiency where the phagocytes lose their ability to kill certain catalase positive pathogens like bacteria and fungi leading to recurrent life-threatening infections and granuloma formation [1].

CGD can be transmitted in an autosomal recessive or x-recessive manner affecting both boys and females as carriers. It is characterized by the development of multiple abscesses in the skin and solid organs that could be associated with the formation of granulomas in the gastrointestinal, genitourinary, and respiratory tract systems with different clinical manifestations [2].

To test the neutrophil's function, the Dihydrorhodamine (DHR) 123 oxidation test by flow cytometry, is the most specific and sensitive. Genetic study to determine the specific mutation is of paramount importance for counseling the parents and to prepare for possible bone marrow transplant which is considered the only curative treatment [3].

The median age at diagnosis of CGD is 2.5 years [3], with only a few cases reported in the neonatal period worldwide. We are reporting this case that presented at the age of 4 days with a neck abscess and was confirmed to be a case of CGD. To our knowledge, it is considered one of the youngest cases to be reported.

## Case presentation

A four-day-old male newborn, born at term by cesarean section, to a multiparous mother with a smooth course of pregnancy with no reported consanguinity or previous similar condition in their siblings or any history of abortions or neonatal deaths due to severe infection. He was discharged from the nursery two days’ postpartum when the mother noticed a small blister on the left base of his neck that was enlarging with associated cellulitis since his admission to our hospital at day 4 of age. There was no fever, signs of respiratory distress, or any associated symptoms nor other skin lesions, masses, or abscesses.

**Figure 1:**
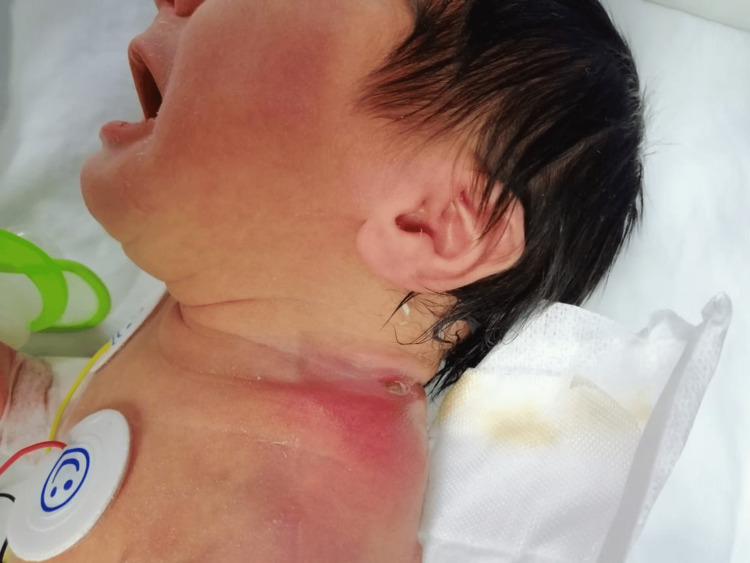
Location of the neck abscess with surrounding inflammatory reaction and the draining sinus opening

**Figure 2:**
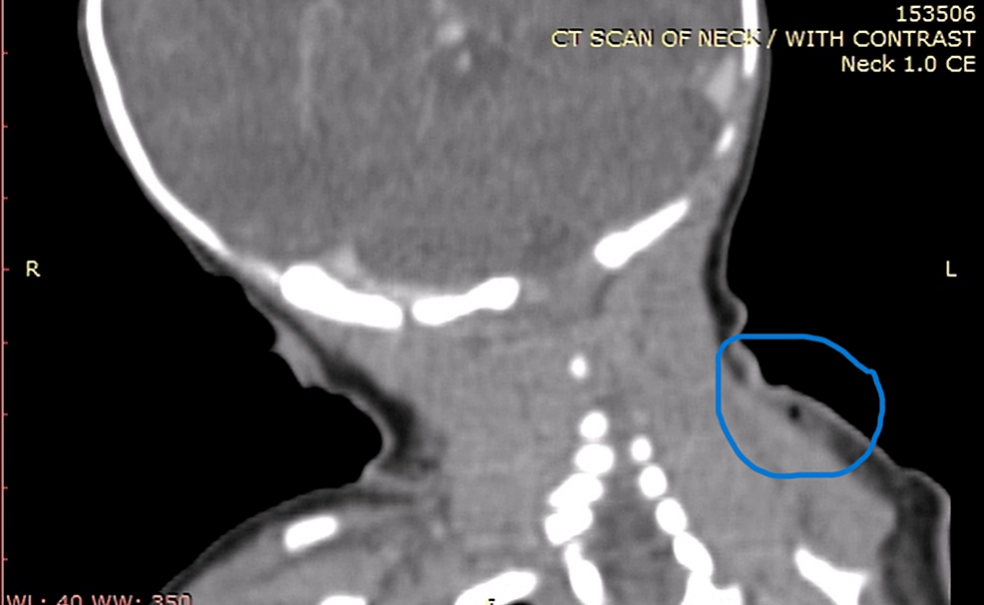
CT scan with Intravenous contrast of the neck showed a 23×13 mm left neck triangle subcutaneous soft tissue edema and gas bubbles favoring cellulitis CT: Computed Tomography

On physical examination: he has normal vital signs with purulent discharge, induration, and overlying cellulitis at the base of the left neck (Figures 1, 2), with no remarkable positive findings on the assessment of other systems including the circumcision site.

The patient was treated by first double antibiotic therapy for the early neonatal sepsis that included ampicillin and Cefotaxime pending the result of the pus culture, which grew on day 2 after admission: An Escherichia coli with an extended spectrum of beta-lactams (ESBL) resistance; so, the antibiotic regimen was changed to include Meropenem and Vancomycin to cover possible methicillin-resistant Staphylococcus aureus.

The surgical team advised to do twice daily sterile dressings of the ruptured abscess. The most relevant routine laboratory findings are included in Table 1.

**Table 1:** Relevant preliminary laboratory findings performed on admission CRP: C-reactive protein, WBC: White blood cells, RBC: Red blood cells, SGPT: Serum glutamic-pyruvic transaminase

Laboratory Values	Patient Results	Normal Reference Range
WBC (x10^e3^/µl)	18.57	4.5-10
Neutrophil Percent (NEUT%)	20.3	40-70
Lymphocyte Percent (LYMPH%)	68.8	20-40
Monocyte Percent (MONO%)	9.3	2-8
Eosinophil Percent (EO%)	1.4	1-4
RBC (x10^e6^/µl)	3.24	4.20-6
Hemoglobin (g/dl)	10.1	13.0-18
Hematocrit (%)	29.6	40-52
Platelets (x10^e3^/µl)	618	150-450
CRP ((mg/dl)	26.0	0-10
Procalcitonin (ng/ml)	1.37	Normal < 0.5
SGPT (IU/L)	6	10-45

A computed tomography (CT) scan of the neck showed a superficial abscess with surrounding reactive cellulitis, extending posteriorly to the left sternocleidomastoid muscle. During his hospital stay, the patient developed an infection of the circumcision site and the remaining umbilical stump.

In view of the multiple skin infections and abscesses starting at an early age, the suspicion of an immunodeficiency disorder was highly considered, we performed a workup that included: a flow cytometry for the Immunologic profile for the main T, B, and NK lymphocyte subpopulations, and in-depth T-cell immunophenotyping and the DHR test.

The result of the DHR test turned out to be positive which confirmed the diagnosis of CGD, but CGD screening for both parents was not performed. The patient received a full course of 21 days of intravenous antibiotics with clinical improvement and he was discharged on oral sulfamethoxazole and trimethoprim and antifungal prophylaxis. Pending the HLA matched donor for possible bone marrow transplant (Table 2).

**Table 2:** Peripheral blood flow cytometry results and interpretation: analysis of the main lymphocyte T, B, and NK populations showed a mild depletion of B cells for age WBC: White blood cells

Results	Percentage	Absolute count	Age Reference Ranges (1 week-1 month)
WBC	100%	11,800/µL	5,000-19,500 Cells/µL
Lymphocytes	55.1%	6,502/ µL	2,500- 16,500 Cells/µL
CD3+	94.6%	6,151/ µL	1,900-8,400 Cells/µL
CD4+	74.9%	4,870/ µL	1,500-6,000 Cells/µL
CD8+	19.4%	1,261/ µL	300-2,700 Cells/µL
CD19+	1.5%	98/ µL	600-1,900 Cells/µL
CD56+CD16+	3.7%	437/ µL	200-1,400 Cells/µL

## Discussion

Our case illustrates the earliest presentation of CGD, a primary immunodeficiency (PID) disorder of phagocytosis dysfunction with impairment of the main function of white blood cells including neutrophils, monocytes, macrophages, and eosinophils that are implicated in the killing process of bacteria and fungi.

CGD was first reported in males, in 1954 as a fatal immunodeficiency disease with recurrent infections associated with a state of hypergammaglobulinemia [4] its incidence varies from one in 200,000 to one in 250,000 live births, according to some studies from the United States and Europe [5]. It was originally supposed to be of an X-linked autosomal recessive inheritance till 1968, when cases of autosomal recessive inheritance were described in girls also, however, the autosomal dominant inheritance was not well-defined yet [6].

Due to impaired neutrophil function, classic infectious presentation involves abscess formation of the skin, liver, and spleen, with the predisposition to develop osteomyelitis, cellulitis, pneumonia, and stomatitis. The most common known bacteria that lead to fatal infections are Staphylococcus aureus and Pseudomonas aeruginosa [7]. However, other organisms are also frequently encountered in CGD patients and may differ according to the geographic location, such as Burkholderia cepacia, Serratia marcescens, Nocardia, Aspergillus, Salmonella, Bacille Calmette-Guérin (BCG), and tuberculosis, all are an important cause of different and severe infections [2].

The suspicion of immunodeficiency disorder could be somewhat problematic in the neonatal period, in the absence of an indicative family history of severe, recurrent, or fatal infections in siblings or relatives, with most of the cases due to de novo mutation. But, whenever we encounter an infant with recurrent, severe, atypical infections due to unusual pathogens, with abnormal or Multiple or recurrent infections with abnormal organisms and poor response to treatment [8]. At this time, the suspicion of immunity disorders should be pursued till the complete identification of such disease, the advancement in genetic screening will be much more helpful nowadays. However, DHR oxidation has recently substituted the oldest Nitroblue Tetrazolium test (NBT), the most familiar diagnostic test for CGD. DHR is preferable because of its relative ease of use and its ability to differentiate the genetic phenotypes of CGD on flow cytometry [9].

The prevention of fatal infections and complications will be the most important step in our management, especially through the implementation of a prophylactic antibiotic regimen, according to each disorder. Lifelong curative treatment is possible with the bone marrow transplant which should be performed ideally as early as possible. The option of early genetic counseling is advisable, in the presence of a positive family history with an identified genetic inheritance like in our case of CGD, which represents one of the youngest reported cases in the medical literature, to the best of our knowledge.

## Conclusions

The primary care physician should have a high level of suspicion whenever he encounters such a severe infection in the neonatal period as illustrated in our case which turned out to be a phagocytosis disorder typical of CGD. The earlier the diagnosis the better will be the therapeutic and preventive approaches, to prevent the possible morbidities associated with the immunodeficiency disorders and to recommend genetic counseling for further pregnancy.
